# Bisphophonates in CKD Patients with Low Bone Mineral Density

**DOI:** 10.1155/2013/837573

**Published:** 2013-12-31

**Authors:** Wen-Chih Liu, Jen-Fen Yen, Cheng-Lin Lang, Ming-Tso Yan, Kuo-Cheng Lu

**Affiliations:** ^1^Department of Internal Medicine, Department of Health, Ministry of Health and Welfare, Chia-Yi Hospital, Chia-Yi, Taiwan; ^2^Department of Internal Medicine, Cardinal Tien Hospital, Yong He Branch, New Taipei, Taiwan; ^3^Division of Nephrology, Department of Medicine, Cathay General Hospital, Taipei, Taiwan; ^4^Division of Nephrology, Department of Medicine, Cardinal Tien Hospital, School of Medicine, Fu-Jen Catholic University, 362 Chung-Cheng Road, Hsin-Tien, New Taipei 231, Taiwan

## Abstract

Patients with chronic kidney disease-mineral and bone disorder (CKD-MBD) have a high risk of bone fracture because of low bone mineral density and poor bone quality. Osteoporosis also features low bone mass, disarranged microarchitecture, and skeletal fragility, and differentiating between osteoporosis and CKD-MBD in low bone mineral density is a challenge and usually achieved by bone biopsy. Bisphosphonates can be safe and beneficial for patients with a glomerular filtration rate of 30 mL/min or higher, but prescribing bisphosphonates in advanced CKD requires caution because of the increased possibility of low bone turnover disorders such as osteomalacia, mixed uremic osteodystrophy, and adynamic bone, even aggravating hyperparathyroidism. Therefore, bone biopsy in advanced CKD is an important consideration before prescribing bisphosphonates. Treatment also may induce hypocalcemia in CKD patients with secondary hyperparathyroidism, but vitamin D supplementation may ameliorate this effect. Bisphosphonate treatment can improve both bone mineral density and vascular calcification, but the latter becomes more unlikely in patients with stage 3-4 CKD with vascular calcification but no decreased bone mineral density. Using bisphosphonates requires considerable caution in advanced CKD, and the lack of adequate clinical investigation necessitates more studies regarding its effects on these patients.

## 1. Introduction

At the onset of chronic kidney disease (CKD), the systemic mineral metabolism and bone composition start to change. This alteration is known as CKD-MBD. The greater the decrease in renal function, the worse the progression of CKD-MBD. CKD-MBD involves serum calcium, serum phosphate, parathyroid hormone (PTH), and vitamin D metabolism derangement, and its main endpoints are altered bone turnover, bone mineralization, bone volume, bone linear growth, bone strength, and vascular/other soft tissue calcification [1].

Those who have CKD-MBD may have another bone problem, osteoporosis, which is the most common metabolic bone disease resulting in fragility fractures. With increasing lifespans, the population with osteoporosis is growing [2, 3], and, in the general population, about 85% of women with osteoporosis have some deterioration of renal function [4]; both are probably attributable to greater age [5]. However, osteoporosis also involves low bone mass, a disarranged microarchitecture, and skeletal fragility, and thus this condition contributes to the risk of fracture, especially in the spine, hip, wrist, humerus, and pelvis [6].

Because fragility fractures, reduced glomerular filtration rate (GFR), and low bone mineral density (BMD) are common in the older population, it can be a challenge to differentiate the cause of a fragility fracture and/or low BMD in elderly CKD (particularly with estimated eGFR <30 mL/minute) patients as either osteoporosis or another bone and mineral disorder related to CKD (e.g., hyperparathyroidism, adynamic bone disease, and osteomalacia) [7] (Table 1). Patients with an age-associated reduction in eGFR to 30 mL/min benefit from oral or intravenous bisphosphonates for osteoporosis [8, 9], but the use of bisphosphonates in CKD requires some caution. Primary management of CKD with low mineral density is essential for understanding the pathogenesis of these two bone disorders and designing a rational approach to treatment and prevention of complications.

## 2. Definition of Osteoporosis

Osteoporosis is a gradual systemic skeletal disease that usually does not cause any symptoms, so many patients do not know that they have it until a bone fracture unexpectedly occurs. In general, osteoporosis is affected by age, sex, body size, ethnicity, family history, sex hormones, and lifestyle.

The structures of the bone consist of the bone matrix, bone cells, and mineral salts. Bone quality is influenced by bone mass, bone density, bone geometry (shape and size), microarchitectural features (cortical or trabecular connections), and molecular elements (collagen type and linkages, bone mineral composition, and crystal orientation). Bone cells include osteoblasts, which form the bone matrix and produce increased bone mass, and osteoclasts, which resorb the bone matrix by secreting acids and digestive enzymes and triggering osteocyte apoptosis and decreased bone mass. In healthy adult bone, the new bone formation will be followed by bone resorption to keep the bone mass in a dynamic balance. However, in osteoporosis, the balance shifts toward bone resorption even though bone mineral and protein are normal, leading to thinning of bone mass and increased fragility [10, 11].

According to 1994 World Health Organization (WHO) criteria, osteoporosis can be defined as a BMD *T*-score < −2.5, as measured by dual-energy X-ray absorptiometry (DXA) at the hip, spine, or forearm [12]. This measure can be applied in both men and women, as well as in younger people with medical problems along with increased risk of low-trauma fracture [13, 14]. This *T*-score is an important diagnostic tool for defining normal density (+1~−1), osteopenia (−1~−2.5), and osteoporosis (<−2.5) in clinical practice. Those with fractures who subsequently undergo BMD testing, however, are more often found to have osteopenia than osteoporosis. One reason is that many other factors not associated with low BMD contribute to fragile bone [15, 16]. Thus, the National Institutes of Health has stated that osteoporosis as “a skeletal disorder characterized by impairment in bone strength predisposing a person to an increased risk of fracture. Bone strength primarily reflects the integration of bone density and bone quality” [17]. This statement does not serve, however, as a diagnostic tool, and, in the general adult population, clinical diagnosis of osteoporosis is made based on one of two conditions: the presence of a low-trauma fracture unrelated to BMD level, or in the absence of a preexisting fracture, a certain level of BMD defined by the standard deviation of the *T*-score [18].

Although the low BMD or fragility fracture is a clear guide for osteoporosis, they do not aid in differentiating cause from metabolic bone diseases that also can lead to fragility fractures. These metabolic bone diseases can be related not only to osteoporosis but also to osteomalacia and adynamic disorders, among others [19]. In addition, DXA checks only area BMD but not bone volumetric BMD, which cannot differentiate cortical from trabecular bone and cannot evaluate bone microarchitecture or bone turnover. To overcome these limitations, some new noninvasive three-dimensional technologies have been developed to evaluate bone microarchitecture, including high-resolution micro-CT and micro-MRI, hip structural analysis, and finite element analysis. Few data with these techniques have been related to CKD patients, however [20].

## 3. Bone Remodeling in CKD

### 3.1. Remodeling

Physiological bone remodeling is a lifelong and highly coordinated process of bone resorption and formation [21, 22]. It involves continuous removal of old bone, replacement with a newly synthesized proteinaceous matrix, and subsequent mineralization of the matrix to form new bone. Therefore, maintaining mineral homeostasis is another essential role of bone remodeling [23]. This resorption and reformation cycle is crucial for repairing bone microfractures and modifying bone structure during stress or other responses to biomechanical forces. Remodeling in the adult bone is not entirely clarified, but the main purposes are removing dead osteocytes, maintaining oxygen and nutrition supply, keeping a suitable level of matrix blood supply, and correcting fatigue damage [24].

The stages of bone remodeling begin with osteoclast resorption and move to reversal, osteoblast maturation, osteoid or unmineralized bone formation, and mineralization to the quiescent stage. In resorption, the osteoclasts gather on the bone surface, releasing acid and hydrolytic enzymes to resorb bone and digest bone minerals and fragments of collagen. The resorption process leads to circulation of free pyridinoline and deoxypyridinoline substances circulating in the blood and eliminated in the urine [25–27]. Bone formation starts from the osteoblasts synthesizing type I collagen and other proteins (e.g., osteocalcin); all of these particles aggregate extracellularly to form osteoid, and then bone is mineralized [22]. The cell membranes of osteoblasts contain alkaline phosphatase. The synthesis of type I collagen is a combination of one alpha-2 and two alpha-1 polypeptide chains to shape a coiled construction known as procollagen [21].

The bone remodeling process occurs through the action of osteoprotegerin (OPG) and the RANKL/RANK (receptor activator of nuclear factor kB; NF-kB ligand/receptor activator of nuclear factor kB; NF-kB) system, which is affected by hormones (PTH, calcitriol, estrogen, and glucocorticoids), cytokines, and interleukins. Increased PTH levels expand the destroyed surface, osteoid surface, and mineralizing surface, which enhance the amount of osteoclasts and osteoblasts [28]. Avbersek-Luznik et al. showed that PTH can enhance bone resorption through a mechanism involving increased osteoblast RANKL synthesis [29]. RANKL/RANK interaction provides essential signals to osteoclast progenitors, leading to osteoclast differentiation, an important step in osteoclastogenesis, and bone resorption. This process can be blocked by OPG, a decoy receptor for RANKL that protects against bone resorption and extensive deterioration [30].

### 3.2. Bone Turnover

Bone strength is determined by its mass, microarchitecture, macrogeometry, and rate of turnover. Bone turnover occurs in both cortical bone and trabecular bone, and the latter has a relatively higher turnover rate [23]. Therefore, this important step of bone turnover is orchestrated by a tightly coupled interplay of osteoclasts and osteoblasts to constantly replace dead bone with new bone [31].

In CKD patients, the effect of high levels of PTH on bone results in the high-turnover bone disease, osteitis fibrosa, with excessive osteoclastic bone resorption and bone marrow fibrosis [32]. In contrast, low-turnover bone disease is common in patients with CKD, especially in dialysis patients, and is characterized by an extremely slow rate of bone formation [19].

Osteomalacia, aluminum-induced bone disease, and adynamic bone disease are low-turnover bone diseases. Osteomalacia is characterized by defective bone mineralization and very slow bone formation rate [33]. This mineralization defect is related to reduced active vitamin D and chronic metabolic acidosis [34]. Aluminum ingestion causes another mineralization defect, owing to reducing both osteoclast resorption and the osteoblast surface, and is associated with low-turnover bone disease. In addition, chronic low-dose aluminum exposure with high intake of vitamin D in dialysis patients reduces PTH synthesis and secretion. These patients may present with adynamic bone disease rather than osteomalacia [35].

## 4. Diagnosis of Osteoporosis or Bone Disease in CKD

### 4.1. Osteoporosis versus Bone Disease in CKD

It is not uncommon that CKD patients develop impairment of bone strength and low-trauma fractures, the latter because of osteoporosis or some other metabolic bone disease associated with CKD. The challenge for clinical practice is to differentiate osteoporosis with CKD-MBD in fracturing patients. However, there are some clues that can be generalized.

First, in early CKD (stages 1 to 3), patients may present with few abnormalities of mineral metabolism and have the same osteoporosis risk factors as the general population. The Kidney Disease: Improving Global Outcomes (K/DIGO) guidelines mention that early CKD metabolic status may involve intermittent hyperphosphatemia, mild increases in PTH, or other bone turnover marker changes without altered bone strength or fragility fractures [36]. Hence, low-trauma fractures in early CKD without abnormalities of vitamin D metabolism and PTH can be diagnosed using the WHO osteoporosis criteria, a *T*-score of −2.5 or lower or fragility fractures, as in the postmenopausal population. In other words, these patients without biochemical abnormalities of CKD-MBD can receive standard treatment [18].

Second, in advanced CKD (stages 4 to 5, 5D), there are no universally accepted criteria for making the diagnosis of osteoporosis [12]. The significant derangements in bone and mineral metabolism may lead to more impairment in bone strength and increased risk for low-trauma fractures. Patients with stage 5D CKD may have a higher risk for fragility fractures than postmenopausal women or elderly men do. These bone fragility conditions are often confounded on DXA with BMD increased due to calcified soft tissue in the path of the X-ray beam or altered bone composition [37]. Therefore, the K/DIGO guidelines do not recommend that patients with stage 3 to 5 CKD receive regular BMD DXA [38].

### 4.2. Diagnosis of Bone Disease in Advanced CKD

As previously described, there are no accredited standards for diagnosing osteoporosis in advanced CKD. For now, the best way to differentiate between osteoporosis and CKD-MBD is bone histomorphometry and/or bone biochemical markers of bone metabolism to describe the bone disease that may be identified for low-trauma fractures in advanced CKD [12]. The K/DIGO guidelines suggest bone biopsy for unexplained fractures, persistent bone pain, unexplained hypercalcemia, high PTH but low alkaline phosphatases, and before therapy with bisphosphonates [38]. To describe the various abnormalities in CKD-MBD, the recent recommendation from the K/DIGO classification of renal metabolic bone disease uses the TMV (turnover: from low to high; mineralization: from normal to abnormal; and bone volume: from low to high) but also does not provide a working diagnosis of osteoporosis [1]. Although many useful radiologic techniques can examine bone microarchitecture to offer the potential for defining turnover, mineralization, and volume noninvasively in advanced kidney disease, they cannot distinguish between renal osteodystrophy and osteoporosis [20, 39–41]. In the future, these noninvasive imaging technologies may lead to methods for connecting turnover, mineralization, and volume to bone strength and open up a totally new way to categorize bone strength [18].

## 5. Bisphosphonates in Advanced CKD

### 5.1. Bisphosphonate Pharmacokinetics

**Figure 1:**
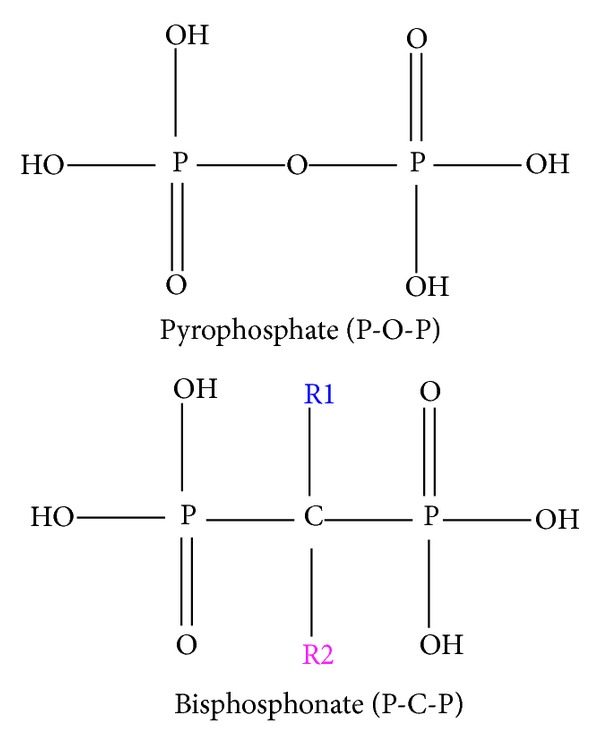
The molecular structure of the bisphosphonates (P-C-P) and pyrophosphates (P-O-P).

Bisphosphonates are standard drugs for osteoporosis and are like pyrophosphate compounds (Figure 1), which are crucial endogenous inhibitors of ectopic mineralization. In postmenopausal women, effective bisphosphonate treatment results in a quantifiable reduction in urinary excretion of N-telopeptide of type I collagen and improvement in bone density [42]. The bone lining cells prevent pyrophosphate from entering the structure by cleaving it with alkaline phosphatase [43]. Because a carbon atom substitutes for the oxygen in pyrophosphate, bisphosphonates are resistant to cleavage by alkaline phosphatase. Therefore, bisphosphonates can enter bone by binding to the mineralized surface [44].

The intestinal absorption of bisphosphonates is low (e.g., 1% for alendronate, ibandronate, and risedronate and 3% to 7% for etidronate [45]) and will be even less when taking medicines without a glass of water or on an empty stomach. When bisphosphonates are absorbed, about 40%–60% enters bone; the rest is excreted unchanged through glomerular filtration with active proximal tubular secretion [46]. Bisphosphonates stack up in bone, with a more than 10-year half-life [47], and are slowly released back into the circulation and taken up again or excreted.

As bone-seeking antireceptive agents [12], bisphosphonates can bind strongly to hydroxyapatite in bone. Bisphosphonates have a carbon atom with two phosphonate groups, forming a P-C-P structure that is a stable analogue of inorganic pyrophosphate [48]. Their effects on hydroxyapatite crystals may influence the overall action of osteoclasts, but the most essential effect is on osteoclasts themselves. During osteoclastic bone resorption, bisphosphonates impair osteoclast cell function by inhibiting enzyme activity [47]. Their mechanism of action, “osteoclast inhibition,” makes them a valuable treatment option in all bone disease associated with increased osteoclast activity. In addition, bisphosphonates can reduce the progression of soft tissue calcification, and recent studies have shown that bisphosphonates have the potential to reduce the progression of vascular calcification [49–51]. Therefore, other roles for bisphosphonates include reducing progression of vascular calcification in CKD.

### 5.2. Mechanisms of Action of Bisphosphonates

Bisphosphonates can be divided into two molecular groups (Table 2): nonnitrogen-containing bisphosphonates (clodronate and etidronate) and nitrogen-containing bisphosphonates (alendronate, ibandronate, pamidronate, risedronate, and zolendronate). When they enter bone and bind with osteoclasts, the latter group is more effective in improving bone strength.

The molecular structure of the nonnitrogen-containing group is very similar to that of pyrophosphate. Thus, these bisphosphonates are incorporated into nonhydrolyzable analogs of ATP, which may limit ATP-dependent enzymes in osteoclasts to block mitochondrial energy production and induce osteoclast apoptosis [52].

**Figure 2:**
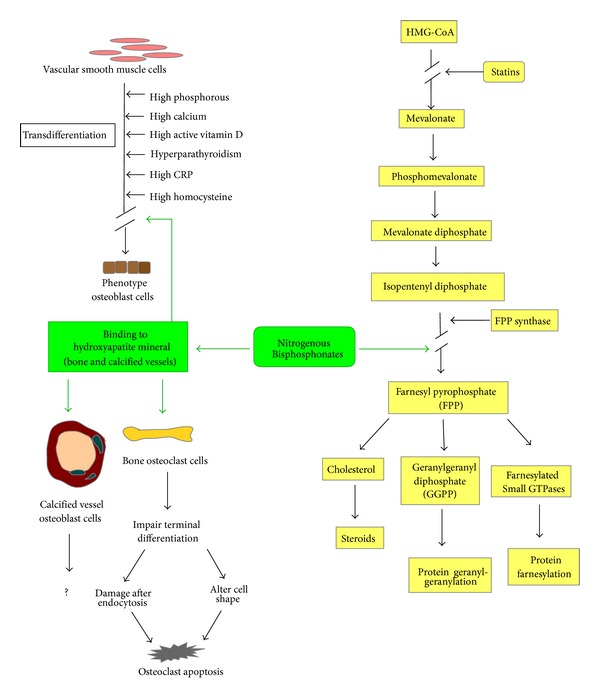
Effects of nitrogenous bisphosphonate on mevalonate metabolism (right) and on the osteoclasts of bone and calcified vessels (left).

The nitrogen-containing bisphosphonates are internalized by endocytosis of osteoclasts and go through other specific biochemical processes to apoptosis (Figure 2). At the same time, they restrict enzymes of the mevalonate pathway and inhibit farnesyl pyrophosphate (FPP) synthase [44]. Furthermore, this inhibition of FPP blocks the prenylation of small GTPase signaling proteins (e.g., Ras, Rho, and Rac), which are essential for osteoclast function and survival [53]. Therefore, the most important key to inhibiting small GTPase binding proteins is to block the biosynthesis of isoprenoid compounds. This capacity can explain the loss of osteoclast activity and function as a result of inhibition of protein prenylation and the disruption of the function of these essential regulatory proteins.

In addition, during bone resorption, the secretion of vacuolar-type proton pumps on the ruffled border of the osteoclast membrane acidifies the space beneath osteoclasts to dissolve bone mineral. When bisphosphonates adsorb to bone mineral, the extracellular bone matrix is destroyed by the action of proteolytic enzymes, and the acidic microenvironment results in the dissolution of the hydroxyapatite bone mineral because bisphosphonates accumulate at the sites of bone resorption where the mineral is most exposed [54, 55]. Osteoclasts, as the most bisphosphonate-attractive cells in bone, take up bisphosphonate separated from bone mineral in the acidified environment beneath osteoclasts. Hence, osteoclasts are exposed to the highest concentrations of free, nonmineral-bound bisphosphonate [48].

Nitrogen-containing bisphosphonates can affect osteoclast functions: terminal differentiation, attachment, endocytosis, cell shape, and apoptosis. In addition to influencing osteoclast function, this type of bisphosphonate can block FPP production in monocytes to gather plenty of isopentenyl diphosphate (IPP). IPP is a bacterial antigen that stimulates T cells to release THF-*α* and INF-*ϒ* and to increase IL-6 and CRP levels. This response is why patients receiving their first bisphosphonate treatment often complain of flu-like symptoms [56].

### 5.3. Bisphosphonate in CKD with Low BMD

In both the general population and patients with stages 1 to 3 CKD, proper treatment with bisphosphonates can prevent fracture [57]. A meta-analysis of postmenopausal women with CKD stages 1 to 3 in nine clinical trials concluded that it is safe to provide bisphosphonates to low-BMD patients without secondary causes or deranged blood levels of calcium, phosphate, PTH, or alkaline phosphatase, and vitamin D abnormalities (laboratory features of CKD-MBD) to reduce fractures [58]. From several prospective studies and clinical trials, oral or intravenous bisphosphonates can offer benefits to patients with aged-related decreased eGFR, down to 30 mL/min [8, 59]. Other researchers as well suggest that it is safe to use bisphosphonates in patients with an eGFR of 30 mL/min or higher [60, 61]. However, these drugs might not be safe when the patients have preexisting renal parenchymal disease (e.g., diabetes) or use other agents that could affect renal function (e.g., nonsteroidal anti-inflammatory drugs). Therefore, when using intravenous bisphosphonates in these specific higher-risk secondary renal disease groups, care is required [18].

The value of bisphosphonate treatment in patients with advanced CKD is vague because suitable data are lacking for CKD stages 4 to 5D (eGFR less than 30 mL/min). Some patients with stages 4 to 5 CKD with fractures and BMD levels in the osteoporotic range may derive benefits from the administration of bisphosphonates [62], as may some dialysis patients with osteoporosis due to gonadal hormone deficiency such as postmenopausal osteoporosis, glucocorticoid-induced osteoporosis, or male osteoporosis [63].

Even though data are limited describing bisphosphonate use in stage 5 CKD, a randomized placebo-controlled study comparing alendronate and placebo in 31 hemodialysis patients proved that hip BMD remained stable after 6 months with alendronate while BMD fell in those treated with placebo [64]. When considering prescribing bisphosphonates to CKD stage 5 patients suffering fragility fractures, renal osteodystrophy must be thoroughly ruled out [65, 66]. Because bone biopsy is not available in most clinics, in patients with CKD-MBD, who already have low bone turnover (e.g., adynamic bone disorder; bone alkaline phosphatase <20 ng/mL and iPTH < 100 pg/mL) [67], the use of bisphosphonates may aggravate clinical symptoms. Therefore, in advanced CKD, a bone biopsy should be considered before providing bisphosphonates, and therapy should involve individual-specific conditions. In patients with advanced CKD, only those who have low BMD and high bone resorption might receive bisphosphonates because these drugs have not been used to prevent fractures in people with normal BMD or with low bone formation.

Current studies show that a 4-hour regular hemodialysis can remove 35% to 40% of the administered bisphosphonate dose, which is close to renal bisphosphonate elimination in normal renal function [68]. But these data are not sufficient evidence for other different bisphosphonates, different dialysis membranes, or the effect of bisphosphonate administration timing related to dialysis timing [69]. Lu et al. (2003) found that pamidronate therapy is associated with reduced plasma iCa levels and increased PTH secretion, resulting in aggravated secondary hyperparathyroidism in postmenopausal hemodialysis patients with SHPT with short-term therapy [70]. However, Huang et al. (2012) [71] demonstrated that, in considering pamidronate treatment in postmenopausal patients with osteoporosis receiving hemodialysis, it might be safer to add calcitriol to prevent increased PTH secretion [71]. Bisphosphonate treatment may induce hypocalcemia, and in CKD patients with secondary hyperparathyroidism, vitamin D supplementation may ameliorate this hypoclacemic effect. Therefore, for patients with early CKD (stages 1–3), nutritional vitamin D (cholecalciferol or ergocalciferol) and calcium should be provided. However, active vitamin D (calcitriol or paricalcitol) could be considered when advanced CKD or dialysis patients receive bisphosphonate treatment.

When managing dialysis patients with osteoporosis, Miller has suggested cutting the bisphosphonate dose to half of the FDA-registered dose for postmenopausal osteoporosis, simply based on limited pharmacokinetic and dialysis data. Moreover, Miller advises limiting treatment to 2-3 years because the reuptake of bisphosphonates might lead to accumulation and unknown bone retention of bisphosphonates in this population [63].

### 5.4. Bisphosphonate and Atherosclerosis with Vascular Calcification

#### 5.4.1. Bisphosphonates and Atherosclerosis

In soft blood vessel tissue, bisphosphonates not only have high affinity for calcium in atherosclerotic deposits but also enter the arterial wall by macrophage phagocytosis [72]. During phagocytosis, bisphosphonates change the function of macrophages to internalize atherogenic LDL cholesterol, consequently transforming LDL into foam cells [73]. Hence, bisphosphonates stimulate macrophage apoptosis through inhibiting intracellular enzymes as well as inhibiting sterol biosynthesis [74]. However, some studies have found that etidronate, pamidronate, and clodronate suppress developing atherosclerosis without influencing cholesterol or lipid levels [74–77].

A study of etidronate therapy showed that four cycles (200 mg daily for 2 wk every 3 months) of etidronate treatments resulted in a remarkable decrease in intima-media thickness. But the selection criteria of participants for those receiving etidronate (on the basis of low BMD) differed from those using placebo (no BMD criteria) [78].

#### 5.4.2. Bisphosphonates and Vascular Calcification

Multiple risk factors cause vascular calcification in CKD patients, such as vascular smooth muscle cell (VSMC) differentiation into osteoblast-like cells, high calcium and phosphorus levels in abnormal bone metabolism, inflammation, low levels of circulating and locally produced inhibitors, impaired renal excretion, and current therapies. It is a highly complicated process that includes a complex interaction of calcification inducers and inhibitors similar to osteogenesis but different from passive mineral deposition [50].

High levels of calcium and phosphate can enhance the activity of VSMC sodium phosphate (NaPi) cotransporters, leading to intracellular phosphate concentration elevation [79, 80]. This result will influence Cbfa-1, an essential controller of cellular differentiation, inducing VSMCs to transdifferentiate into the osteoblast phenotype cell [62].

In the 1970s, some results indicated that bisphosphonates would be able to inhibit vascular calcification in both humans and non-human animals [81, 82]. Bisphosphonates do inhibit the expression of TNF-*α*, which can induce osteogenic transdifferentiation processes and calcium deposition in atheromatous injury of rabbit aorta [77].

Several studies have shown that CKD 4-5 patients usually have adynamic bone disease in bone biopsy samples along with some degree of arterial calcification [83, 84], even with the progression of coronary artery calcification [85]. However, Toussaint et al. [86] found that 51 patients with stage 3-4 CKD treated with alendronate for 18 months in clinical trials did not show inhibition of progression of vascular calcification compared with placebo. Therefore, in CKD patients with low BMD, bisphosphonate treatment may improve both the BMD and vascular calcification, but in CKD patients with vascular calcification but no decreased BMD, bisphosphonate treatment is unlikely to do so [86]. In addition, PTH levels had a meaningful increase with alendronate versus placebo (+3.2 pmol/L [95% CI, 0.8–5.5]; *P* = 0.009) at 18 months [86].

During low bone turnover, serum phosphate does not easily enter the bone, and the bone therefore cannot buffer elevation of the phosphate burden any longer. Cannata-Andia and colleagues suggested that bone inhibition and vascular calcifications create a vicious cycle [87]. Sclerostin, an inhibitor of Wnt signaling, is released from transdifferentiated VSMCs as a defensive response that aims to block the Wnt pathway to reduce the mineralization in the vascular tissue [88]. It not only slows the transformation of VSMCs into osteoblasts but also may overflow to the circulation and lessen bone formation, which in turn weakens the ability of bone to absorb phosphate. Hence, bisphosphonates would worsen bone low turnover, then result in high serum phosphate, which would further increase VSMC transformation to osteoblast-like cells [87, 89].

The mechanism by which bisphosphonates inhibit vascular calcification remains unclear. They may inhibit bone resorption, reduce serum calcium and phosphate, and limit their deposition in the vascular wall [90] or their ability to influence the activity of the VSMC NaPi cotransporter. Other case studies indicate that both etidronate [91] and pamidronate are useful [92] in treating calciphylaxis (uremic arteriolopathy), which is a rare but life-threatening complication of CKD.

### 5.5. Renal Complications of Bisphosphonates

Monitoring proteinuria in patients when administering bisphosphonates is recommended because several groups have mentioned that bisphosphonates damage kidney tissues [93, 94]. As an oncology medicine, bisphosphonates produce direct nephrotoxicity, particularly when used at high dosage [95]. Administering high-dose IV pamidronate can bring on collapsing focal glomerulosclerosis and resulting nephrotic proteinuria [93]. The FDA suggests adjusting the dosage of zoledronic acid according to the baseline creatinine clearance and administering the infusion over 15 minutes [63]. A fast IV of bisphosphonates has been associated with acute renal failure complication [96–98]. Treatment of pamidronate may cause nephrotic syndrome with renal impairment [94] or acute tubular necrosis with consequent acute renal impairment [99].

Markowitz et al. analyzed bone biopsies from six patients who developed renal failure after treatment with zolendronate and identified tubular atrophy, interstitial fibrosis, interstitial inflammation, and mild-to-moderate vascular disease as the main mechanisms of renal damage [98]. Lower doses of bisphosphonate only rarely induce clinically significant nephrotoxicity [100].

## 6. Conclusions

CKD patients often suffer from osteoporosis. To diagnosis osteoporosis in CKD patients, WHO criteria and/or low-trauma fractures can be widely used for stages 1 to 3 CKD, but there is no consensus regarding the criteria for the diagnosis of osteoporosis in stage 4, 5, or 5D CKD. Patients in stages 4, 5, and 5D CKD have a high prevalence of other metabolic bone diseases and CKD-MBD, which increases difficulties in managing these more complex situations, and the WHO criteria or fragility fractures cannot be used for the diagnosis of osteoporosis in these stages.

That bisphosphonates in advanced CKD may be associated with low bone turnover is uncertain because they decrease bone turnover in a preexisting low bone turnover state, which can influence bone in different ways and result in more or less cardiovascular disease.

Bone biopsy in advanced CKD is a priority consideration before prescribing bisphosphonates in these patients because these medicines will increase the possibility of worsening bone turnover, osteomalacia, and mixed uremic osteodystrophy, and aggravate hyperparathyroidism. In the dialysis population, bisphosphonates should be carefully considered as therapeutic agents and with very specific requirements: carefully defined osteoporosis, dose adjustment considerations, and limited exposure time.

Thus, the bisphosphonates should be used with caution in the CKD patient population and treatment tailored to each individual. Currently, the role of bisphosphonates in BMD and vascular calcification in CKD population remains unclear. It must be stated strongly that most of these compounds are substantially renally excreted and thus tend to accumulate significantly in plasma and bone, with prolonged and often excessive skeletal actions. In patients with early CKD (stages 1–3), nutritional vitamin-D (cholecalciferol or ergocalciferol) and calcium should be provided. However, in patients with advanced CKD or dialysis active vitamin-D (calcitriol or paricalcitol) could be considered. Diagnosis and treatment of low BMD in CKD, and any impact on associated tendencies to vascular calcification, remain challenging and underresearched. Because the current evidence for this population is insufficient, further and detailed research is required to better delineate how to prescribe these medicines appropriately.

## Figures and Tables

**Table 1:** Bone diseases may associate with fragility fractures (low trauma fractures).

Name of bone diseases	General metabolic bone diseases	Chronic kidney disease-mineral bone disease (CKD-MBD)
Adynamic bone disease (including aluminum bone disease)		Yes
Amyloid bone disease	Yes (AL-amyloidosis.)	Yes (*β*_2_-microglobulin)
Mixed uremic osteoporosis		Yes
Osteitis fibrosa cystica (severe)	Yes (primary hyperparathyroidism)	Yes (secondary hyperparathyroidism)
Osteogenesis imperfecta	Yes	

Osteomalacia		
Vitamin D-related	Yes	Yes
Nonvitamin D-related		
Chronic metabolic acidosis	Yes	Yes
Phosphate depletion	Yes	Yes

Osteoporosis		
Primary osteoporosis	Yes	Yes
Secondary osteoporosis (all causes)		
Chronic liver disease	Yes	
Hypogonadism or premature menopause	Yes	
Inflammatory bowel disease	Yes	
Malabsorption	Yes	
Steroid-induced osteoporosis	Yes	

Pathologic fractures (malignancies)	Yes	

Paget's disease	Yes	

**Table 2:** Two classes of bisphosphonates.

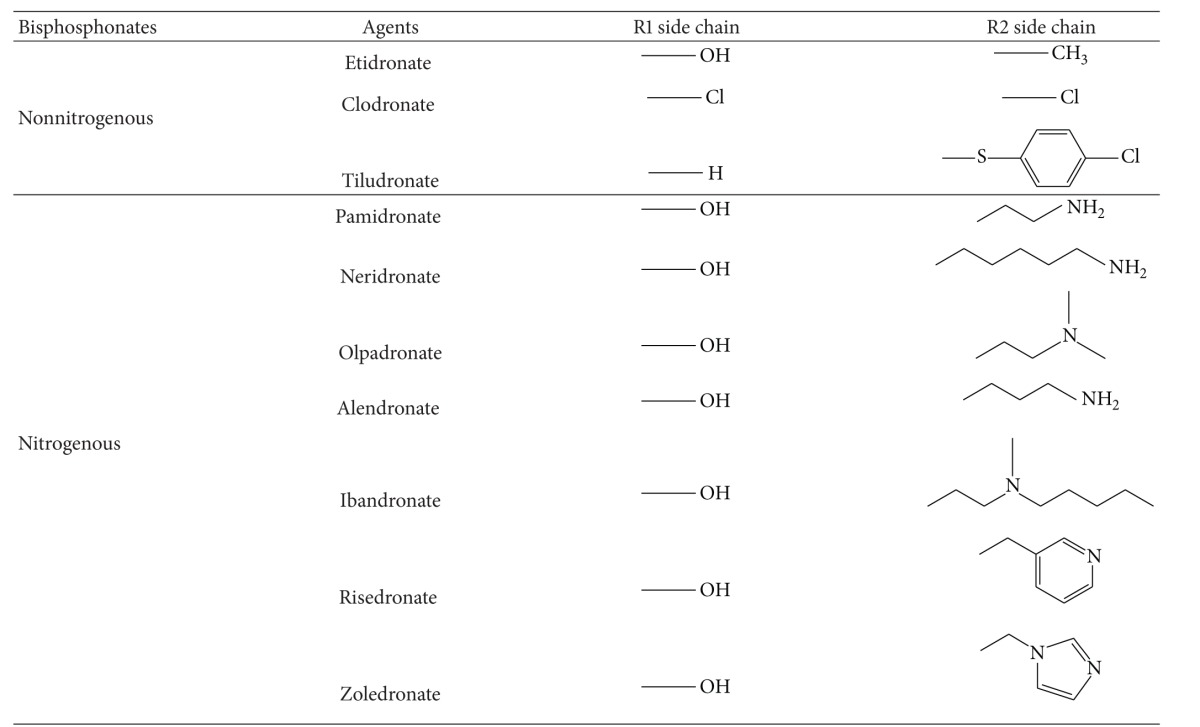

The bisphosphonates are analogous to that of the naturally occurring pyrophosphates, with two-side chains (R1 and R2) attached to the carbon core. The R1 side chain determines bone-binding affinity, and the R2 side chain determines interception potency.

## References

[1] Moe S, Drüeke T, Cunningham J (2006). Definition, evaluation, and classification of renal osteodystrophy: a position statement from Kidney Disease: improving Global Outcomes (KDIGO). *Kidney International*.

[2] Melton LJ (2003). Epidemiology worldwide. *Endocrinology and Metabolism Clinics of North America*.

[3] Burge R, Dawson-Hughes B, Solomon DH, Wong JB, King A, Tosteson A (2007). Incidence and economic burden of osteoporosis-related fractures in the United States, 2005–2025. *Journal of Bone and Mineral Research*.

[4] Klawansky S, Komaroff E, Cavanaugh PF (2003). Relationship between age, renal function and bone mineral density in the US population. *Osteoporosis International*.

[5] Hsu C-Y, Cummings SR, McCulloch CE, Chertow GM (2002). Bone mineral density is not diminished by mild to moderate chronic renal insufficiency. *Kidney International*.

[6] Riggs BL, Melton LJ (1995). The worldwide problem of osteoporosis: insights afforded by epidemiology. *Bone*.

[7] Gal-Moscovici A, Sprague SM (2007). Osteoporosis and chronic kidney disease. *Seminars in Dialysis*.

[8] Eisman JA, Civitelli R, Adami S (2008). Efficacy and tolerability of intravenous ibandronate injections in postmenopausal osteoporosis: 2-year results from the DIVA study. *Journal of Rheumatology*.

[9] Black DM, Delmas PD, Eastell R (2007). Once-yearly zoledronic acid for treatment of postmenopausal osteoporosis. *The New England Journal of Medicine*.

[10] Wilkins CH, Birge SJ (2005). Prevention of osteoporotic fractures in the elderly. *American Journal of Medicine*.

[11] Benhamou C-L (2007). Effects of osteoporosis medications on bone quality. *Joint Bone Spine*.

[12] Miller PD (2009). Diagnosis and treatment of osteoporosis in chronic renal disease. *Seminars in Nephrology*.

[13] Baim S, Binkley N, Bilezikian JP (2008). Official positions of the international society for clinical densitometry and executive summary of the 2007 ISCD position development conference. *Journal of Clinical Densitometry*.

[14] Assessment of Fracture Risk its Application to Screening for Postmenopausal Osteoporosis (1994). *Report of a WHO Study Group*.

[15] Bouxsein ML (2004). Non-invasive measurements of bone strength: promise and peril. *Journal of Musculoskeletal Neuronal Interactions*.

[16] Seeman E (2008). Bone quality: the material and structural basis of bone strength. *Journal of Bone and Mineral Metabolism*.

[17] Nih Consensus Development Panel on Osteoporosis Prevention, D., and Therapy (2001). Osteoporosis prevention, diagnosis, and therapy. *The Journal of the American Medical Association*.

[18] Miller PD (2009). Fragility fractures in chronic kidney disease: an opinion-based approach. *Cleveland Clinic Journal of Medicine*.

[19] Martin KJ, González EA (2007). Metabolic bone disease in chronic kidney disease. *Journal of the American Society of Nephrology*.

[20] Wehrli FW, Leonard MB, Saha PK, Gomberg BR (2004). Quantitative high-resolution magnetic resonance imaging reveals structural implications of renal osteodystrophy on trabecular and cortical bone. *Journal of Magnetic Resonance Imaging*.

[21] Baim S, Miller PD (2009). Assessing the clinical utility of serum CTX in postmenopausal osteoporosis and its use in predicting risk of osteonecrosis of the jaw. *Journal of Bone and Mineral Research*.

[22] Calvo MS, Eyre DR, Gundberg CM (1996). Molecular basis and clinical application of biological markers of bone turnover. *Endocrine Reviews*.

[23] Clarke B (2008). Normal bone anatomy and physiology. *Clinical Journal of the American Society of Nephrology*.

[24] Seeman E (2003). The structural and biomechanical basis of the gain and loss of bone strength in women and men. *Endocrinology and Metabolism Clinics of North America*.

[25] Taylor AK, Lueken SA, Libanati C, Baylink DJ (1994). Biochemical markers of bone, turnover for the clinical assessment of bone metabolism. *Rheumatic Disease Clinics of North America*.

[26] Hanson DA, Weis MAE, Bollen A-M, Maslan SL, Singer FR, Eyre DR (1992). A specific immunoassay for monitoring human bone resorption: quantitation of type I collagen cross-linked N-telopeptides in urine. *Journal of Bone and Mineral Research*.

[27] Garnero P, Gineyts E, Arbault P, Christiansen C, Delmas PD (1995). Different effects of bisphosphonate and estrogen therapy on free and peptide-bound bone cross-links excretion. *Journal of Bone and Mineral Research*.

[28] Ritz E, Stefanski A, Rambausek M (1995). The role of the parathyroid glands in the uremic syndrome. *American Journal of Kidney Diseases*.

[29] Avbersek-Luznik I, Balon BP, Rus I, Marc J (2005). Increased bone resorption in HD patients: is it caused by elevated RANKL synthesis?. *Nephrology Dialysis Transplantation*.

[30] Aoki S, Honma M, Kariya Y (2010). Function of OPG as a traffic regulator for RANKL is crucial for controlled osteoclastogenesis. *Journal of Bone and Mineral Research*.

[31] Ducy P, Desbois C, Boyce B (1996). Increased bone formation in osteocalcin-deficient mice. *Nature*.

[32] Spaulding CM, Young G (1997). Osteitis fibrosa cystica and chronic renal failure. *Journal of the American Podiatric Medical Association*.

[33] Gonzalez EA, Martin KJ (1992). Aluminum and renal osteodystrophy: a diminishing clinical problem. *Trends in Endocrinology and Metabolism*.

[34] Coen G, Manni M, Addari O (1995). Metabolic acidosis and osteodystrophic bone disease in predialysis chronic renal failure: effect of calcitriol treatment. *Mineral and Electrolyte Metabolism*.

[35] Cannata-Andia JB, Harrington JT, Martinez-Maldonado M (1998). Hypokinetic azotemic osteodystrophy. *Kidney International*.

[36] Moe SM, Drüeke T (2008). Improving global outcomes in mineral and bone disorders. *Clinical Journal of the American Society of Nephrology*.

[37] Gordon PL, Frassetto LA (2010). Management of osteoporosis in CKD Stages 3 to 5. *American Journal of Kidney Diseases*.

[38] Kidney Disease: Improving Global Outcomes (KDIGO) CKD-MBD Work Group (2009). KDIGO clinical practice guideline for the diagnosis, evaluation, prevention, and treatment of Chronic Kidney Disease-Mineral and Bone Disorder (CKD-MBD). *Kidney International Supplements*.

[39] Nickolas TL, Leonard MB, Shane E (2008). Chronic kidney disease and bone fracture: a growing concern. *Kidney International*.

[40] Genant HK, Lang TF, Engelke K (1996). Advances in the noninvasive assessment of bone density, quality, and structure. *Calcified Tissue International*.

[41] Roschger P, Paschalis EP, Fratzl P, Klaushofer K (2008). Bone mineralization density distribution in health and disease. *Bone*.

[42] Hung KC, Huang CY, Liu CC (2012). Serum bone resorption markers after parathyroidectomy for renal hyperparathyroidism: correlation analyses for the cross-linked N-telopeptide of collagen I and tartrate-resistant acid phosphatase. *The Scientific World Journal*.

[43] Whyte MP, Greenberg CR, Salman NJ (2012). Enzyme-replacement therapy in life-threatening hypophosphatasia. *The New England Journal of Medicine*.

[44] Russell RGG, Watts NB, Ebetino FH, Rogers MJ (2008). Mechanisms of action of bisphosphonates: similarities and differences and their potential influence on clinical efficacy. *Osteoporosis International*.

[45] Razzaque MS, Lanske B (2007). The emerging role of the fibroblast growth factor-23-klotho axis in renal regulation of phosphate homeostasis. *Journal of Endocrinology*.

[46] Troehler U, Bonjour JP, Fleisch H (1975). Renal secretion of diphosphonates in rats. *Kidney International*.

[47] Lin JH (1996). Bisphosphonates: a review of their pharmacokinetic properties. *Bone*.

[48] Russell RGG, Rogers MJ, Frith JC (1999). The pharmacology of bisphosphonates and new insights into their mechanisms of action. *Journal of Bone and Mineral Research*.

[49] Negri AL (2005). Vascular calcifications in chronic kidney disease: are there new treatments?. *Current Vascular Pharmacology*.

[50] Toussaint ND, Kerr PG (2007). Vascular calcification and arterial stiffness in chronic kidney disease: implications and management. *Nephrology*.

[51] Tamura K, Suzuki Y, Matsushita M (2007). Prevention of aortic calcification by etidronate in the renal failure rat model. *European Journal of Pharmacology*.

[52] Frith JC, Mönkkönen J, Blackburn GM, Russell RGG, Rogers MJ (1997). Clodronate and liposome-encapsulated clodronate are metabolized to a toxic ATP analog, adenosine 5′-(*β*,*γ*-dichloromethylene) triphosphate, by mammalian cells in vitro. *Journal of Bone and Mineral Research*.

[53] Coxon FP, Rogers MJ (2003). The role of prenylated small GTP-binding proteins in the regulation of osteoclast function. *Calcified Tissue International*.

[54] Masarachia PJ, Weinreb M, Balena R, Rodan GA (1996). Comparison of the distribution of 3H-alendronate and 3H-etidronate in rat and mouse bones. *Bone*.

[55] Azuma Y, Sato H, Oue Y (1995). Alendronate distributed on bone surfaces inhibits osteoclastic bone resorption in vitro and in experimental hypercalcemia models. *Bone*.

[56] Bukowski JF, Dascher CC, Das H (2005). Alternative bisphosphonate targets and mechanisms of action. *Biochemical and Biophysical Research Communications*.

[57] Cunningham J (2007). Pathogenesis and prevention of bone loss in patients who have kidney disease and receive long-term immunosuppression. *Journal of the American Society of Nephrology*.

[58] Miller PD, Roux C, Boonen S, Barton IP, Dunlap LE, Burgio DE (2005). Safety and efficacy of risedronate in patients with age-related reduced renal function as estimated by the Cockcroft and Gault method: a pooled analysis of nine clinical trials. *Journal of Bone and Mineral Research*.

[59] Miller PD (2008). Anti-resorptives in the management of osteoporosis. *Best Practice and Research*.

[60] Perazella MA, Markowitz GS (2008). Bisphosphonate nephrotoxicity. *Kidney International*.

[61] Boonen S, Sellmeyer DE, Lippuner K (2008). Renal safety of annual zoledronic acid infusions in osteoporotic postmenopausal women. *Kidney International*.

[62] Toussaint ND, Elder GJ, Kerr PG (2009). Bisphosphonates in chronic kidney disease; balancing potential benefits and adverse effects on bone and soft tissue. *Clinical Journal of the American Society of Nephrology*.

[63] Miller PD (2007). Is there a role for bisphosphonates in chronic kidney disease?. *Seminars in Dialysis*.

[64] Wetmore JB, Benet LZ, Kleinstuck D, Frassetto L (2005). Effects of short-term alendronate on bone mineral density in haemodialysis patients. *Nephrology*.

[65] Miller PD (2008). The role of bone biopsy in patients with chronic renal failure. *Clinical Journal of the American Society of Nephrology*.

[66] Ferreira MA (2000). Diagnosis of renal osteodystrophy: when and how to use biochemical markers and non-invasive methods; when bone biopsy is needed. *Nephrology Dialysis Transplantation*.

[67] Ureña P, Hruby M, Ferreira A, Ang KS, de Vernejoul M-C (1996). Plasma total versus bone alkaline phosphatase as markers of bone turnover in hemodialysis patients. *Journal of the American Society of Nephrology*.

[68] Bergner R (2013). Bisphosphonate therapy in renal osteodystrophy—a review. *Journal of Nephrology*.

[69] Buttazzoni M, Rosa Diez GJ, Jager V, Crucelegui MS, Algranati SL, Plantalech L (2006). Elimination and clearance of pamidronate by haemodialysis. *Nephrology*.

[70] Lu K-C, Yeung L-K, Lin S-H, Lin Y-F, Chu P (2003). Acute effect of pamidronate on PTH secretion in postmenopausal hemodialysis patients with secondary hyperparathyroidism. *American Journal of Kidney Diseases*.

[71] Huang CY, Zheng CM, Wu CC, Lo L, Lu KC, Chu P (2012). Effects of pamidronate and calcitriol on the set point of the parathyroid gland in postmenopausal hemodialysis patients with secondary hyperparathyroidism. *Nephron Clinical Practice*.

[72] Rogers MJ, Xiong X, Ji X (1997). Inhibition of growth of Dictyostelium discoideum amoebae by bisphosphonate drugs is dependent on cellular uptake. *Pharmaceutical Research*.

[73] Ylitalo R, Monkkonen J, Ylä-Herttuala S (1997). Effects of liposome-encapsulated bisphosphonates on acetylated LDL metabolism, lipid accumulation and viability of phagocyting cells. *Life Sciences*.

[74] Ylitalo R (2000). Bisphosphonates and atherosclerosis. *General Pharmacology*.

[75] Kramsch DM, Aspen AJ, Rozler LJ (1981). Atherosclerosis: prevention by agents not affecting abnormal levels of blood lipids. *Science*.

[76] Zhu B-Q, Sun Y-P, Sievers RE, Isenberg WM, Moorehead TJ, Parmley WW (1994). Effects of etidronate and lovastatin on the regression of atherosclerosis in cholesterol-fed rabbits. *Cardiology*.

[77] Ylitalo R, Oksala O, Ylä-Herttuala S, Ylitalo P (1994). Effects of clodronate (dichloromethylene bisphosphonate) on the development of experimental atherosclerosis in rabbits. *Journal of Laboratory and Clinical Medicine*.

[78] Koshiyama H, Nakamura Y, Tanaka S, Minamikawa J (2000). Decrease in carotid intima-media thickness after 1-year therapy with etidronate for osteopenia associated with type 2 diabetes. *Journal of Clinical Endocrinology and Metabolism*.

[79] Chen NX, O’Neill KD, Duan D, Moe SM (2002). Phosphorus and uremic serum up-regulate osteopontin expression in vascular smooth muscle cells. *Kidney International*.

[80] Giachelli CM (2003). Vascular calcification: in vitro evidence for the role of inorganic phosphate. *Journal of the American Society of Nephrology*.

[81] Fleisch HA, Russell RG, Bisaz S, Mühlbauer RC, Williams DA (1970). The inhibitory effect of phosphonates on the formation of calcium phosphate crystals in vitro and on aortic and kidney calcification in vivo. *European Journal of Clinical Investigation*.

[82] Russell RG, Smith R, Bishop MC, Price DA (1972). Treatment of myositis ossificans progressiva with a diphosphonate. *The Lancet*.

[83] London GM, Marty C, Marchais SJ, Guerin AP, Metivier F, De Vernejoul M-C (2004). Arterial calcifications and bone histomorphometry in end-stage renal disease. *Journal of the American Society of Nephrology*.

[84] Tomiyama C, Carvalho AB, Higa A, Jorgetti V, Draibe SA, Canziani MEF (2010). Coronary calcification is associated with lower bone formation rate in CKD patients not yet in dialysis treatment. *Journal of Bone and Mineral Research*.

[85] Barreto DV, Barreto FDC, de Carvalho AB (2008). Association of changes in bone remodeling and coronary calcification in hemodialysis patients: a prospective study. *American Journal of Kidney Diseases*.

[86] Toussaint ND, Lau KK, Strauss BJ, Polkinghorne KR, Kerr PG (2010). Effect of alendronate on vascular calcification in CKD stages 3 and 4: a pilot randomized controlled trial. *American Journal of Kidney Diseases*.

[87] Cannata-Andia JB, Roman-Garcia P, Hruska K (2011). The connections between vascular calcification and bone health. *Nephrology Dialysis Transplantation*.

[88] Viaene L, Behets GJ, Claes K (2013). Sclerostin: another bone-related protein related to all-cause mortality in haemodialysis?. *Nephrology, Dialysis, Transplantation*.

[89] Ott SM (2012). Bisphosphonate safety and efficacy in chronic kidney disease. *Kidney International*.

[90] Persy V, De Broe M, Ketteler M (2006). Bisphosphonates prevent experimental vascular calcification: treat the bone to cure the vessels?. *Kidney International*.

[91] Shiraishi N, Kitamura K, Miyoshi T (2006). Successful treatment of a patient with severe calcific uremic arteriolopathy (calciphylaxis) by etidronate disodium. *American Journal of Kidney Diseases*.

[92] Monney P, Nguyen Q-V, Perroud H, Descombes E (2004). Rapid improvement of calciphylaxis after intravenous pamidronate therapy in a patient with chronic renal failure. *Nephrology Dialysis Transplantation*.

[93] Markowitz GS, Appel GB, Fine PL (2001). Collapsing focal segmental glomerulosclerosis following treatment with high-dose pamidronate. *Journal of the American Society of Nephrology*.

[94] Sauter M, Jülg B, Porubsky S (2006). Nephrotic-range proteinuria following pamidronate therapy in a patient with metastatic breast cancer: mitochondrial toxicity as a pathogenetic concept?. *American Journal of Kidney Diseases*.

[95] Bergner R, Diel IJ, Henrich D, Hoffmann M, Uppenkamp M (2006). Differences in nephrotoxicity of intravenous bisphosphonates for the treatment of malignancy-related bone disease. *Onkologie*.

[96] Pfister T, Atzpodien E, Bohrmann B, Bauss F (2005). Acute renal effects of intravenous bisphosphonates in the rat. *Basic and Clinical Pharmacology and Toxicology*.

[97] Chang JT, Green L, Beitz J, Tarassoff P, Hei Y-J, Maladorno D (2003). Renal failure with the use of zoledronic acid. *The New England Journal of Medicine*.

[98] Markowitz GS, Fine PL, Stack JI (2003). Toxic acute tubular necrosis following treatment with zoledronate (Zometa). *Kidney International*.

[99] Banerjee D, Asif A, Striker L, Preston RA, Bourgoignie JJ, Roth D (2003). Short-term, high-dose pamidronate-induced acute tubular necrosis: the postulated mechanisms of bisphosphonate nephrotoxicity. *American Journal of Kidney Diseases*.

[100] Cunningham J (2007). Bisphosphonates in the renal patient. *Nephrology Dialysis Transplantation*.

